# Large interlayer spacing Nb_4_C_3_T_*x*_ (MXene) promotes the ultrasensitive electrochemical detection of Pb^2+^ on glassy carbon electrodes†

**DOI:** 10.1039/d0ra04377j

**Published:** 2020-06-29

**Authors:** P. Abdul Rasheed, Ravi P. Pandey, Tricia Gomez, Michael Naguib, Khaled A. Mahmoud

**Affiliations:** Qatar Environment and Energy Research Institute (QEERI), Hamad Bin Khalifa University (HBKU), Qatar Foundation P. O. Box 34110 Doha Qatar kmahmoud@hbku.edu.qa; Department of Physics and Engineering Physics, Tulane University New Orleans LA USA

## Abstract

A Nb_4_C_3_T_*x*_ (MXene)-modified glassy carbon electrode was used for the electrochemical detection of Pb^2+^ ions in aqueous media. The sensing platform was evaluated by anodic stripping analysis after optimizing the influencing factors such as pH, deposition potential, and time. The large interlayer spacing, high *c* lattice parameter and higher conductivity of Nb_4_C_3_T_*x*_ compared to other MXenes enhance the electrochemical detection of Pb^2+^. The developed sensor can reach a detection limit of 12 nM at a potential ∼−0.6 V. Additionally, the developed sensor showed promising selectivity in the presence of Cu^2+^ and Cd^2+^, and stability for at least 5 cycles of continuous measurements with good repeatability. This work demonstrates the potential applications of Nb_4_C_3_T_*x*_ towards the development of effective electrochemical sensors.

## Abstract 2

Large interlayer spacing Nb_4_C_3_T_*x*_ (MXene) promotes the ultrasensitive electrochemical detection of Pb^2+^ on glassy carbon electrodes

## Graphical abstract

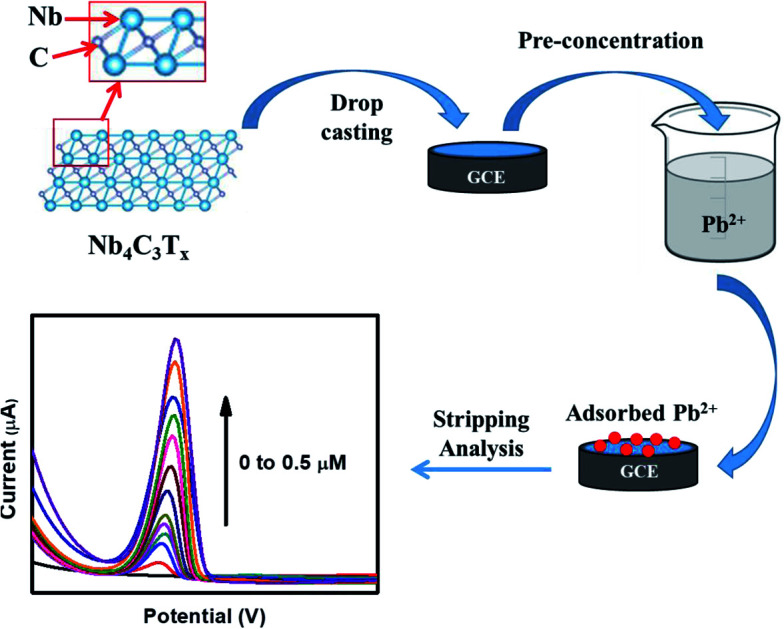

## Introduction

Lead (Pb) is a common heavy metal, used in a variety of industrial processes and anthropogenic activities.^1^ Pb^2+^ ions are known for their extremely harmful biological toxicity through enzyme inhibition and induction of oxidative stress and can cause chronic damage to several human body systems, including kidneys, gastrointestinal system, nervous system, and reproductive system.^2–4^ Moreover, Pb contamination poses a serious health and environmental hazard due to its high accumulation and low clearance rate at contaminated sites.^5,6^ The maximum level of Pb^2+^ in drinking water set by the United States Environmental Protection Agency (EPA) is 15 μg L^−1^ (72 nM),^7^ while the World Health Organization (WHO) limit for the blood Pb^2+^ level is 100 μg L^−1^ (483 nM).^5,8^

Various techniques are used for the detection of Pb^2+^ in water including atomic absorption/fluorescence spectrometry, optical emission spectrometry, inductively coupled plasma mass spectrometry, and chemical or optical sensors.^9–11^ Despite their reliability, the operations and maintenance associated with these methods are tedious, costly, and not suitable for on-site monitoring. Electrochemical techniques are inexpensive, selective, highly sensitive and effective alternative for the detection of various toxic substances and heavy metals.^12–14^ Moreover, electrochemical methods are characterized by their portability, easy operation, quick analysis time, and low maintenance and instrumentation costs.^8,15^ Stripping voltammetry (especially anodic stripping) have been used as the sensitive and powerful electrochemical technique for the detection of heavy metal ions.^1,9^ Different carbon nanomaterials such as carbon nanotubes, graphene, and its composite materials have been used as the sensing platform for sensitive Pb^2+^ detection.^16–20^

The 2D transition metal carbides and carbonitrides (MXenes) have attracted broad attention with unique physicochemical properties.^21–24^ The large lateral size with few nanometer thickness, embedded with good hydrophilicity, and activated metallic hydroxide sites render MXenes as promising materials for environmental remediation applications.^25–28^ MXene surfaces is negatively charged due to its surface functional groups, which facilitate the adsorption of several toxic heavy metals and emerging contaminants.^27,29^ In addition, MXenes nanosheets having strong trapping power to small cations, due to less inter planner distance (<2 Å).^30^ It was reported that Ti_3_C_2_T_*x*_ which is the most studied MXene, is an efficient adsorbent for the gold, lead and chromium cations.^30^ In addition, the intercalation of different cations with various sizes and charges are possible between Ti_3_C_2_T_*x*_ layers.^30^ After alkalization intercalation of Ti_3_C_2_T_*x*_, alk-MXene (Ti_3_C_2_(OH/ONa)_*x*_F_2−*x*_) exhibits superior sorption behavior for Pb^2+^ in presence of high levels of interfering cations such as Ca^2+^ and Mg^2+^.^31^ Recently, alkalization-intercalated Ti_3_C_2_T_*x*_ modified electrode displayed enhanced electrochemical response towards the detection of Cd^2+^, Pb^2+^, Cu^2+^ and Hg^2+^.^32^ The alkalization process increases the *c* lattice parameter of Ti_3_C_2_T_*x*_ from 19.741 Å to 26.187 Å and the alkalization process results in unique morphology and alteration in surface chemistry. This leads to enhanced electrochemical responses for alkalization-intercalated Ti_3_C_2_T_*x*_ towards the heavy metal detection in comparison with the Ti_3_C_2_T_*x*_.

Nb_4_C_3_T_*x*_ is another member of the MXenes family, prepared by etching of Al from the Nb_4_AlC_3_ MAX phase.^33,34^ Recently, Nb_4_C_3_T_*x*_ have been explored in a number of applications, including dye adsorption,^35^ energy storage devices,^23,36–38^ hematopoietic recovery,^33^ photothermal tumor eradication,^22^ supercapacitors,^39^ and photocatalytic hydrogen production.^40^ Even though, the electrochemical performance of Nb_4_C_3_T_*x*_ has not been widely explored towards sensing applications.

In this paper, we evaluate the electrochemical performance of Nb_2_CT_*x*_ and Nb_4_C_3_T_*x*_ on the glassy carbon electrode (GCE) and their application as sensing platform for the detection of Pb^2+^ in the aqueous media. To the best of our knowledge, this is the first report discusses the application of Nb_4_C_3_T_*x*_ as electrochemical sensor for heavy metals.

## Experimental

### Materials

Phosphate buffer (PB) solution, NaOH, H_2_SO_4_, K_3_[Fe(CN)_6_], K_4_[Fe(CN)_6_]·3H_2_O, sodium acetate, acetic acid, lead nitrate, copper sulfate and cadmium acetate were purchased from Sigma Aldrich. The MAX phases of Nb_2_CT_*x*_ and Nb_4_C_3_T_*x*_ (Nb_2_AlC and Nb_4_AlC_3_ respectively) were prepared by mixing powders of niobium (Alfa Aesar, 99.98%, −325 mesh), aluminum (Alfa Aesar, 99.9%, −325 mesh), and carbon (Alfa Aesar, 99%, 7–11 micron) at different ratios, followed by heating under an argon (Ar) flow. The detailed procedure is given in the ESI† and in the ref. 34 and 37.

### Synthesis of Nb_2_CT_*x*_ and Nb_4_C_3_T_*x*_

The synthesis of multilayered Nb_2_CT_*x*_ (ML-Nb_2_CT_*x*_) and Nb_4_C_3_T_*x*_ (ML-Nb_4_C_3_T_*x*_) MXenes were done by hydrofluoric acid (HF) etching of Al layers from MAX phases Nb_2_AlC and Nb_4_AlC_3_ respectively. The Nb_2_AlC or Nb_4_AlC_3_ powders were stirred for 96 h at 40 °C after immersing in 50% HF aqueous solution. The resulting reaction mixture were washed 5 to 6 times using DI water and centrifuged at 3500 rpm to separate the ML-MXenes as settled powders from the supernatants. The resulting ML-MXenes were washed using ethanol, and dried at 30 °C under flow of argon. The delaminated Nb_2_CT_*x*_ (DL-Nb_2_CT_*x*_) and Nb_4_C_3_T_*x*_ MXenes (DL-Nb_4_C_3_T_*x*_) flakes were prepared by probe sonication (Cole Parmer, Ultrasonic Processor, 60% amplitude, 750 watt) of ML-Nb_2_CT_*x*_ and ML-Nb_4_C_3_T_*x*_ MXenes (100 mg) in 5 mL of degassed DI water at 20 °C, under a flow of Ar gas for 1 h, followed by freeze-drying.

### Characterization

The morphology of prepared DL-Nb_2_CT_*x*_ and DL-Nb_4_C_3_T_*x*_ MXenes were characterized by scanning electron microscopy (SEM), using a FEI Quanta 650 FEG. The transmission electronic microscopy (TEM) was performed by using FEI Talos F200×. The ethanol dispersions of DL-Nb_2_CT_*x*_ and DL-Nb_4_C_3_T_*x*_ were mounted on a lacey Formvar carbon-coated Cu grid for TEM analysis. Bruker D8 Advance X-ray diffractometer with Cu-Kα radiation (*λ* = 1.54056 Å) was used to record X-ray diffractograms.

### Fabrication of DL-Nb_2_CT_*x*_ and DL-Nb_4_C_3_T_*x*_ modified electrodes and electrochemical analysis

Prior to experiments, GCE was polished with alumina powder followed by sonication in a copious amount of ethanol and distilled water. 0.2 mg of Nb_2_CT_*x*_/Nb_4_C_3_T_*x*_ was dissolved in 1 mL of distilled water and homogeneous suspension was made by sonication for 1 min. Then, 6 μL of this suspension was deposited onto GCE and dried at room temperature for overnight under inert atmosphere. CHI760E electrochemical work station (CHI, Texas, USA) was used to conduct all electrochemical measurements with a three electrode system. The three electrode system consist of a modified GCE as the working electrode, Pt wire as the counter electrode, and Ag/AgCl in saturated KCl as the reference electrode. Cyclic voltammetry (CV) were performed at a scan rate of 100 mV s^−1^ in the 0.1 M PB solution (pH 7) and in the solution of 0.1 M KCl with 10 mM [Fe(CN)_6_]^3−/4−^. Electrochemical impedance spectroscopy (EIS) measurements were performed at a potential of 10 mV in the 100 kHz to 0.1 Hz frequency range.

### Stripping voltammetry analysis

Square wave anodic stripping voltammetry (SWASV) measurements were used to detect Pb^2+^ in acetate buffer solution (0.1 M, pH 5.0) containing different concentration Pb^2+^. The pre concentration step was performed at −1.2 V for 150 s while stirring the electrolyte solution. SWASV voltammograms were recorded after an equilibration period of 15 s, in the potential range from −0.8 V to 0 V with square wave potential scan having 4 mV increment potential, 25 mV amplitude and 50 Hz frequency. After each anodic stripping measurement, a desorption step was performed at a potential of 0.8 V for 100 s under stirring to remove the residual heavy metal ions on the electrode surface. For interference measurements, the pre concentration step was carried out at the potential of −1.2 V for 150 s while string the electrolyte solution containing Cd^2+^ and Cu^2+^ (5 times concentration than Pb^2+^) followed by recording SWASV voltammograms in the potential range from −1.2 V to 0 V. Error bars shows the standard deviation for three repetitive measurements in each experiment.

## Results and discussion

### Material characterization

**Fig. 1:**
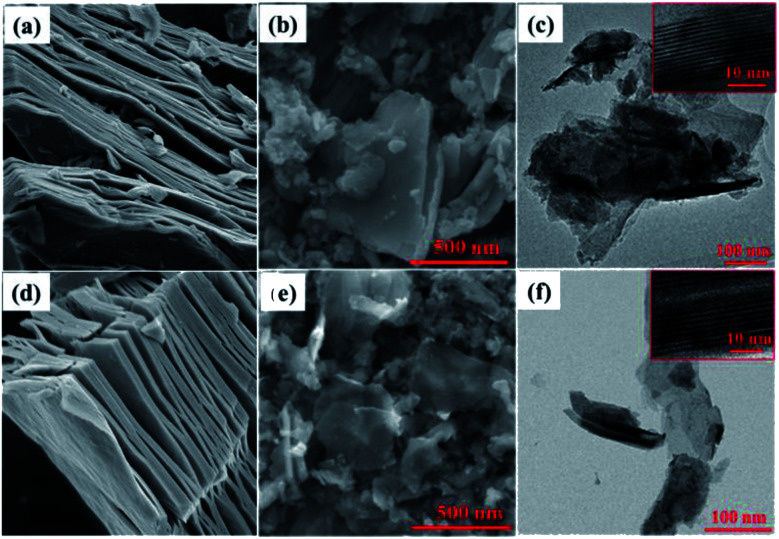
SEM image of (a) ML-Nb_2_CT_*x*_ (b) DL-Nb_2_CT_*x*_ (d) ML-Nb_4_C_3_T_*x*_ and (e) DL-Nb_4_C_3_T_*x*_. TEM image of (c) DL-Nb_2_CT_*x*_ and (f) DL-Nb_4_C_3_T_*x*_. HR-TEM image of DL-Nb_2_CT_*x*_ and DL-Nb_4_C_3_T_*x*_ are shown in the inset of (c) and (f) respectively.

DL-Nb_2_CT_*x*_ and DL-Nb_4_C_3_T_*x*_ MXenes nanosheets were prepared by acid etching of Al layer using HF aqueous solution from their corresponding MAX phases as described in the Experimental section, followed by sonication and freeze drying. SEM images in (Fig. 1(a and c)) describe the typical accordion-like structure in both multi-layered (ML)-Nb_2_CT_*x*_ and ML-Nb_4_C_3_T_*x*_ (Fig. 1(a and c)). After probe sonication, DL-Nb_2_CT_*x*_ and DL-Nb_4_C_3_T_*x*_ showed similar wrinkled sheet-like structure (Fig. 1(b and d)). Energy-dispersive spectroscopy (EDS) confirmed the presence of fluorine, oxygen, carbon and niobium elements in both MXenes (Fig. S1†). The TEM images revealed electron transparent single or few sheets with an average of 200–400 nm sheet size (Fig. 1(c and f)). In addition, the high resolution TEM (HR-TEM) images of DL-Nb_2_CT_*x*_ and DL-Nb_4_C_3_T_*x*_ shown in the inset shows the *d*-spacing ∼11.5 Å and ∼15 Å, respectively.^36^ The XRD patterns of ML-Nb_2_CT_*x*_, DL-Nb_2_CT_*x*_, ML-Nb_4_C_3_T_*x*_ and DL-Nb_4_C_3_T_*x*_ are given in Fig. S2.† After delamination the intensity of (002) peaks were increased while intensity of other peaks decreased. The strong characteristic peak (002) in both DL-Nb_2_CT_*x*_ (at 2theta of 7.87°) and DL-Nb_4_C_3_T_*x*_ (at 2theta of 5.94°) confirmed the successful delamination and preparation of DL-MXenes.^23,35^ DL-Nb_2_CT_*x*_ has a smaller *c* lattice parameter (c-LP) of 22.44 Å as compared to 29.70 Å for DL-Nb_4_C_3_T_*x*_, as calculated from (002) peak position. The corresponding interlayer distance for DL-Nb_2_CT_*x*_ was 11.22 Å and 14.85 Å for DL-Nb_4_C_3_T_*x*_, which is in a good agreement with the TEM results. The interlayer spacing in DL-Nb_4_C_3_T_*x*_ were higher than that of DL-Nb_2_CT_*x*_, which could explain the interplanar distance increases with carbide blocks (*n*) in each MXene layer of M_*n*+1_X_*n*_T_*x*_. A 14.85 Å spacing of DL-Nb_4_C_3_T_*x*_ is larger than most studied MXenes.^41^ As far as the electrochemical performance is concerned, larger interlayer space allows faster adsorption and intercalation of ions, and it enhances ion diffusion and charge transport of the electrolyte.^42^

### Electrochemical characterization of Nb_2_CT_*x*_ and Nb_4_C_3_T_*x*_

**Scheme 1:**
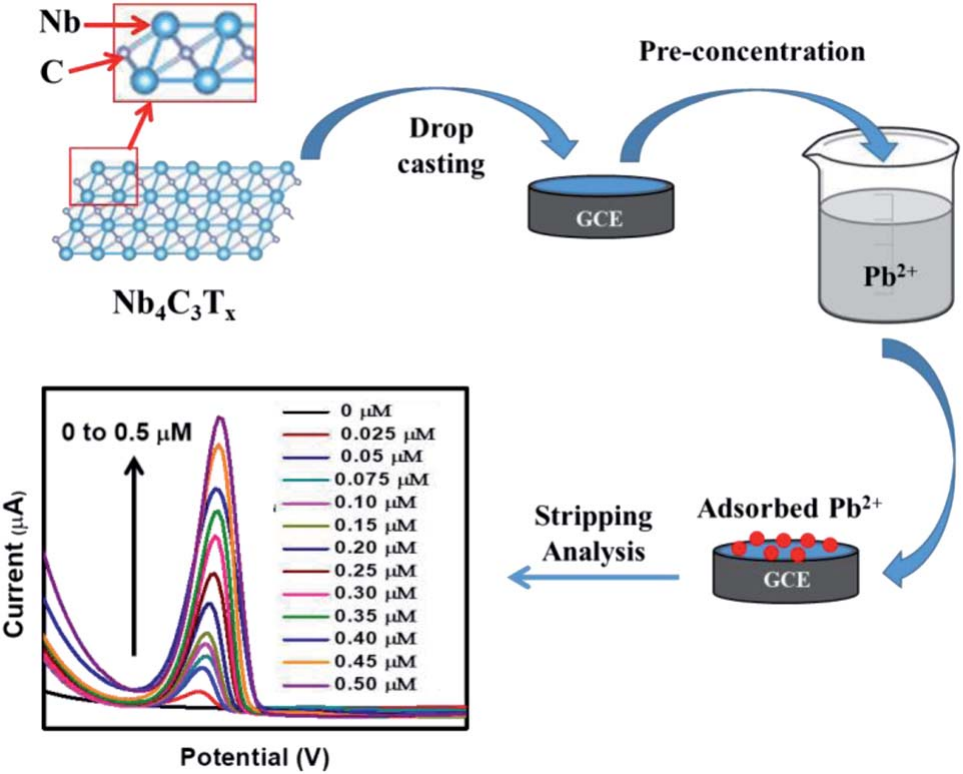
The electrode fabrication and development of Nb_4_C_3_T_*x*_ modified sensor for Pb^2+^ detection.

**Fig. 2:**
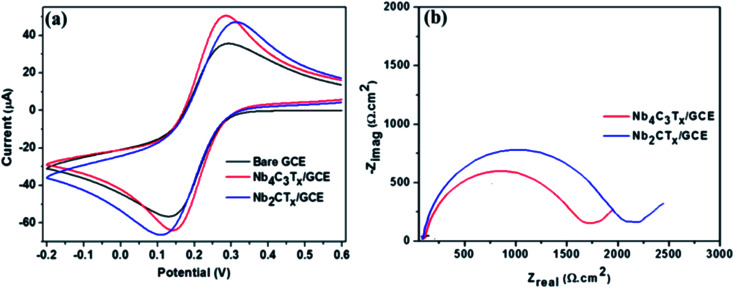
(a) CVs of bare GCE, Nb_2_CT_*x*_/GCE and Nb_4_C_3_T_*x*_/GCE. (b) Nyquist plots of Nb_2_CT_*x*_/GCE and Nb_4_C_3_T_*x*_/GCE. Frequency range: 0.1 Hz to 10 kHz. The experiments were performed in the solution of 0.1 M KCl with 10 mM [Fe(CN)_6_]^3−/4−^.

The fabrication of Nb_4_C_3_T_*x*_ modified GCE and the development of Nb_4_C_3_T_*x*_ modified sensor for Pb^2+^ detection is given in Scheme 1. CV and EIS analysis were used to investigate the electrochemical behaviour of Nb_2_CT_*x*_ and Nb_4_C_3_T_*x*_ modified electrodes in an aqueous solution containing ferrocyanide/ferricyanide redox couple solution. As observed in Fig. 2(a), well-defined redox peaks were observed for all the electrodes and these peaks can be attributed to the reversible redox behaviour of [Fe(CN)_6_]^3−/4−^. The Δ*E*_p_ values for Nb_2_CT_*x*_/GCE and Nb_4_C_3_T_*x*_/GCE were 197 mV and 141 mV, respectively and the lowest Δ*E*_p_ value of Nb_4_C_3_T_*x*_/GCE indicating highest electron transfer kinetics than Nb_2_CT_*x*_/GCE. In addition, the Δ*I*_p_ values for Nb_2_CT_*x*_/GCE and Nb_4_C_3_T_*x*_/GCE were 114.51 mV and 110.10 mV respectively. The higher value of Δ*I*_p_ again confirmed the highest electron transfer kinetics of Nb_4_C_3_T_*x*_/GCE than Nb_2_CT_*x*_/GCE. The electrochemical active surface area was calculated by using the Randles–Sevcik equation and it has a value of 0.574 × 10^−3^ cm^2^ and 0.621 × 10^−3^ cm^2^ for Nb_2_CT_*x*_ and Nb_4_C_3_T_*x*_ respectively (see ESI†).^43^ The Nyquist plot for Nb_2_CT_*x*_/GCE and Nb_4_C_3_T_*x*_/GCE is given in Fig. 2(b). The charge transfer resistance (*R*_ct_) parameter was obtained after fitting the Nyquist plot and was used to evaluate the electron-transfer kinetics of the redox couple at the electrode interface.^44^ The *R*_ct_ values obtained for Nb_2_CT_*x*_/GCE and Nb_4_C_3_T_*x*_/GCE were (2142 ± 24) Ω and (1732 ± 19) Ω respectively by fitting with R(Q[RW]) Randles equivalent circuit. The resistivity is lowest for Nb_4_C_3_T_*x*_/GCE and hence Nb_4_C_3_T_*x*_ is having the highest conductivity than Nb_2_CT_*x*_ which can be justified by the higher ‘*n*’ value of Nb_4_C_3_T_*x*_ (*n* = 3) than Nb_2_CT_*x*_ (*n* = 1).^45^ From the CV and EIS analysis, it was found that Nb_4_C_3_T_*x*_ having highest electrochemical activity.

### Stripping behaviour of Pb^2+^ and optimization of experimental parameters

**Fig. 3:**
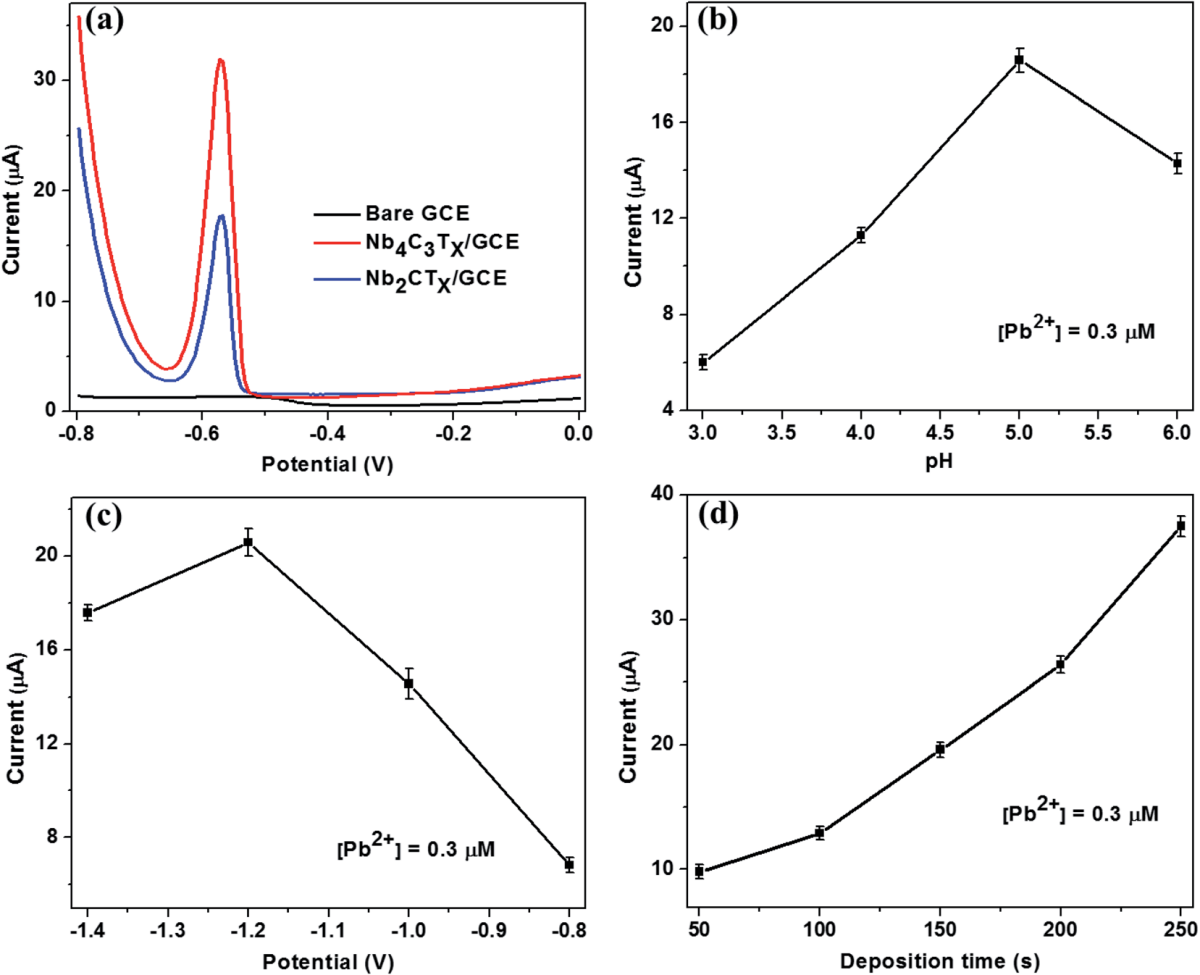
(a) SWASV responses for bare GCE, Nb_2_CT_*x*_/GCE and Nb_4_C_3_T_*x*_/GCE in presence of 0.5 μM of Pb^2+^ in 0.5 M acetate buffer at pH 5, deposition potential of −1.2 V and deposition time of 150 s. Optimization of experimental parameters. (b) pH, (c) deposition potential, and (d) deposition time towards the stripping current of Nb_4_C_3_T_*x*_/GCE.

The SWASV response of the bare GCE, Nb_2_CT_*x*_/GCE and Nb_4_C_3_T_*x*_/GCE were analyzed for the detection of Pb^2+^ ions using acetate buffer solution containing 0.5 μM Pb^2+^. Compared with the bare GCE, well defined stripping peaks at around −0.58 V were observed for the Nb_2_CT_*x*_/GCE and Nb_4_C_3_T_*x*_/GCE and the peak current is highest for Nb_4_C_3_T_*x*_/GCE than Nb_2_CT_*x*_/GCE (Fig. 3(a)). The highest response for Nb_4_C_3_T_*x*_ can be attributed to large interlayer spacing, high *c* lattice parameter value than Nb_2_CT_*x*_ which is evident from TEM and XRD measurements. In addition, it was established that the resistivity of Nb_4_C_3_T_*x*_ is lower than Nb_2_CT_*x*,_^45^ which corresponds to the higher conductivity of Nb_4_C_3_T_*x*_ as evident from electrochemical analysis (Fig. 2). Hence, Nb_4_C_3_T_*x*_ has been selected as the sensing platform for the sensitive detection of Pb^2+^.

The optimum conditions for highly sensitive Pb^2+^ detection were evaluated by changing the critical parameters such as pH, deposition time and potential. The impact of pH on the stripping current was studied from 3.0 to 6.0 (Fig. 3(b)). The peak current of Pb^2+^ was increased with increasing the pH from 3.0 to 5.0 and then decreased at pH = 6. The presence of [Nb–O]–H^+^ groups favours the ion exchange behaviour of Nb_4_C_3_T_*x*_ and this behaviour increases with the pH which results in the strengthening of stripping current.^32^ The decrease in peak currents at pH 6 could be attributed to the hydrolysis of cations results in the formation of more Pb(OH)_2_, which inhibits the further accumulation of Pb^2+^. Considering the maximum observed stripping peak current, pH 5 was selected as optimal for subsequent experiments.

Deposition potential and time are also critical factors for stripping analysis to detect heavy metal ions. The deposition potential was varied from −1.4 to −0.8 V and the resulting stripping currents increases with negative potential until −1.2 V (Fig. 3(c)). A reduction in the current response was observed at deposition potential lower than −1.2 V. This might be due to occurrence of more hydrogen evolution in the acetate buffer. Hence, the deposition potential of −1.2 V was selected as optimal for further experiments. The deposition time was varied from 50 to 250 s and the stripping peak currents response was evaluated (Fig. 3(d)). The stripping peak current have increased linearly with the deposition time increase. A deposition time of 150 s was selected for subsequent experiments considering the concession between short measurement time, high sensitivity and good reproducibility favoured for practical applications.

### Quantitative detection of Pb^2+^

**Fig. 4:**
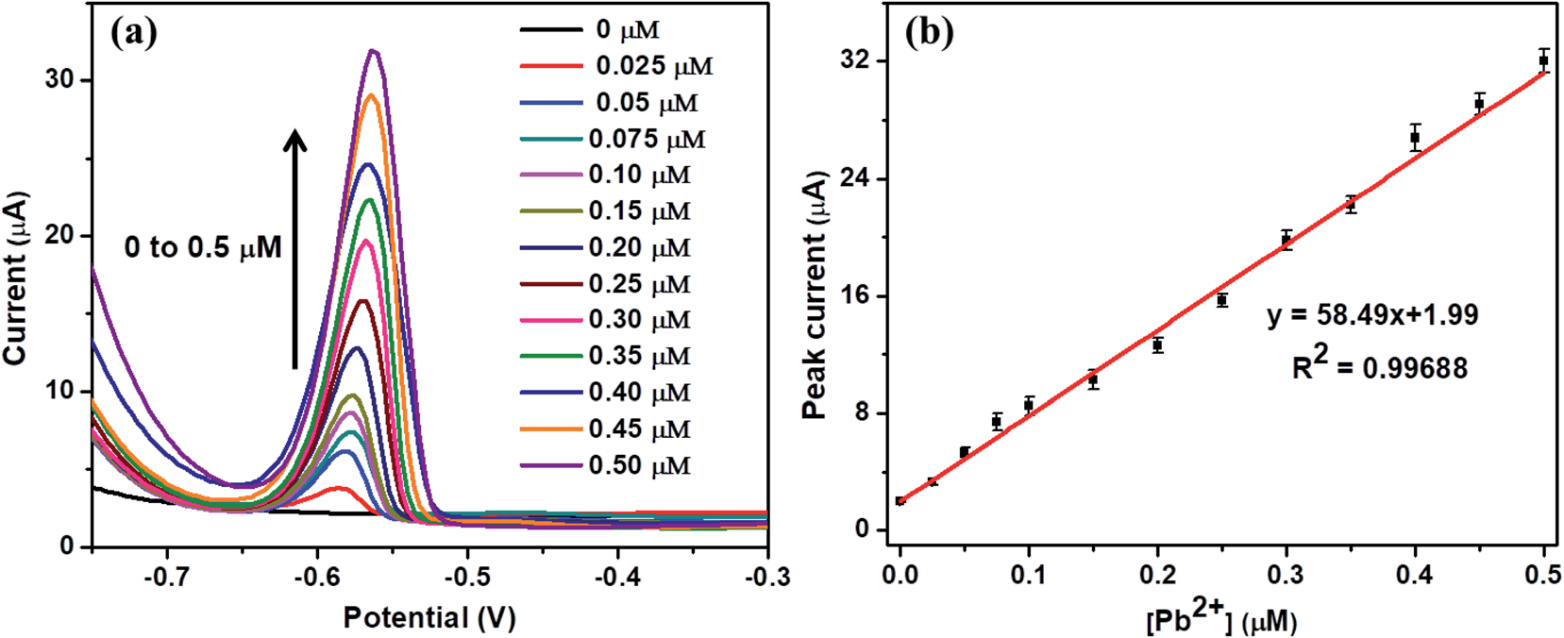
(a) SWASV response of the Nb_4_C_3_T_*x*_/GCE for in presence of Pb^2+^ from 0 to 0.5 μM. (b) The calibration plot of peak current *vs.* Pb^2+^ concentrations.

Under the optimal conditions, the quantitative detection of Pb^2+^ was performed by SWASV on Nb_4_C_3_T_*x*_/GCE. Fig. 4(a) shows the SWASV responses at different concentrations from 0 to 0.5 μM of Pb^2+^. The stripping peak currents increases with increasing the Pb^2+^ concentration and a good linear relationship was observed in the concentration range from 0.025 μM to 0.5 μM. There was no response for the developed sensor when the Pb^2+^ concentration was less than 0.025 μM and this concentration can be regarded as limit of quantification of the sensor. The corresponding calibration plot is given in Fig. 4(b), by plotting the peak current *vs.* Pb^2+^ concentration. The calibration plot equation was represented as *i* (μA) = 58.49[Pb^2+^] + 1.13, with 0.99688 as correlation coefficient (*R*^2^). The limit of detection (LOD) was calculated as 12 nM (S/N = 3), which is much lower (or comparable) than the similar kind of sensors for Pb^2+^ detection (refer Table 1). In a similar work with alkaline intercalated Ti_3_C_2_T_*x*_ MXene as platform for electrochemical detection of heavy metals, the stripping analysis showed a detection limit of 32 nM with linear range of 0.10–0.55 μM and this response is significantly higher than bare Ti_3_C_2_T_*x*_. The analytical performance of Nb_4_C_3_T_*x*_ modified GCE is higher compared to bare Ti_3_C_2_T_*x*_ as well as alkaline intercalated Ti_3_C_2_T_*x*_. The analytical performance of Nb_4_C_3_T_*x*_ modified GCE was compared with other similar reported electrodes (summarized in Table 1) and it showed that the Nb_4_C_3_T_*x*_ modified GCE exhibited a wider range, promising detection limit and enhanced sensitivity. The large interlayer spacing and high *c* lattice parameter of Nb_4_C_3_T_*x*_ (in comparison with Nb_2_CT_*x*_) have allowed for the adsorption of larger amount of Pb^2+^ between the sheets as well as the higher conductivity of Nb_4_C_3_T_*x*_ have improved the electrochemical response on the electrode surface. This strategy can be easily implemented into screen printed electrodes for practical and portable applications considering the versatile sensor fabrication by drop-casting Nb_4_C_3_T_*x*_ on the electrode followed by drying.^46^

**Table 1:** The analytical performance of different 2D materials used for Pb^2+^ detection

Electrode	Detection technique	Detection limit	Linear range	Ref.
l-Cys/AuNPs/NG/GCE	SWV	56 nM	1–80 μM	1
Bismuth modified exfoliated graphite	SWASV	53 nM	1.0–250 μM	9
MWCNT/P1,5-DAN/Pt	SWASV	2.1 μM	4 to 150 μM	16
MWCNT/5-Br-PADAP/GCE	SWASV	100 nM	0.9 to 114.6 μM	17
G/PANI/PS nanoporous fiber/SPCE	SWASV	3.30 μM	10-500 μM	18
l-Cys-rGO/GCE	DPASV	215 nM	0.4 to 1.2 μM	19
Nafion/G/PANI nanocomposite/SPE	SWASV	100 nM	1–300 μM	20
Alk-Ti_3_C_2_/GCE	SWASV	32 nM	0.10–0.55 μM	32
Nb_4_C_3_T_*x*_/GCE	SWASV	12 nM	0.025–0.5 μM	This work

### Selectivity, stability and repeatability

**Fig. 5:**
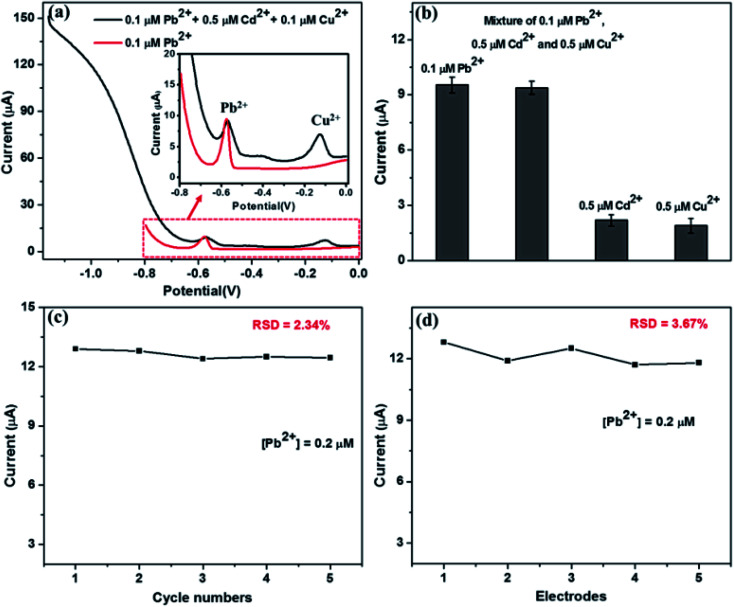
(a) SWASV response of the Nb_4_C_3_T_*x*_/GCE for Pb^2+^ (0.1 μM) in presence of Cd^2+^ (0.5 μM) and Cu^2+^ (0.5 μM). (b) Selectivity plot-SWASV response of the Nb_4_C_3_T_*x*_/GCE at −0.6 V for different heavy metals. (c) The stability of Nb_4_C_3_T_*x*_/GCE – SWASV responses for 5 repetitive cyclic measurements with 0.2 μM Pb^2+^. (d) The repeatability of Nb_4_C_3_T_*x*_/GCE – SWASV responses for 5 different electrodes with 0.2 μM Pb^2+^.

The selectivity of the Nb_4_C_3_T_*x*_/GCE was analyzed in the presence of Cu^2+^ and Cd^2+^ as inferring agents in 5 fold excess along with Pb^2+^ ion. The SWASV was performed under the optimum conditions. It is found that no or negligible change in the peak current of Pb^2+^ in presence of interfering ions. The peak for Cu^2+^ at around −0.1 V,^32^ is present in the SWASV curve as shown in Fig. 5(a). The peak for Cd^2+^ at around −0.8 V,^32^ is not clearly visible; however, the current response is higher in the particular range where the peak for Cd^2+^ normally visible. Fig. 5(b) shows the current response for the individual metal ions (Pb^2+^, Cd^2+^, Cu^2+^) and their mixture at −0.58 V. The peak current was almost the same for Pb^2+^ alone and in the mixture with other interfering ions. These is no current response at particular potential of −0.58 V for the electrolyte solution containing Cd^2+^ or Cu^2+^. These results confirmed the insignificant impact of interfering metal ions and hence the selectivity of the sensor at around −0.58 V.

The stability of the Nb_4_C_3_T_*x*_/GCE was investigated after 5 repetitive measurements using the same electrode and electrolyte containing 0.2 μM Pb^2+^. After every measurement, a desorption step at a potential of 0.8 V was performed before the next electrodeposition steps. It is found that the SWASV response was highly reproducible with RSD value of 2.34 (Fig. 5(c)). In addition, the stripping current of Nb_4_C_3_T_*x*_/GCE for Pb^2+^ was measured after keeping the electrode at 4 °C for one week and the stripping current obtained was 94.3% of the initial current with RSD of 3.15. These results confirmed the stability of the Nb_4_C_3_T_*x*_/GCE electrode towards the detection of Pb^2+^. The reproducibility analysis of the sensor was carried out by using five identical Nb_4_C_3_T_*x*_/GCE electrodes for the detection of Pb^2+^ using similar procedures. The SWASV responses for five different electrodes with 0.2 μM Pb^2+^ showing good repeatability between different electrodes with RSD value of 3.67 (Fig. 5(d)).

### Practical applications

To evaluate the practical applications of the developed Nb_4_C_3_T_*x*_/GCE sensor, the response of the sensor has been measured after spiking the Pb^2+^ in bottled and tap water samples. The SWASV response of the sensor has been measured and the results are given in Table 2. The sensor exhibited a promising recovery between 95% and 102% with a relative standard deviation of 1.8–3.0%. From these results, it was confirmed that the developed sensor can be used for the detection of Pb^2+^ from samples.

**Table 2:** Recovery of Pb^2+^ in drinking water and tap water using the Nb_4_C_3_T_*x*_/GCE sensor

Sample	Pb^2+^ added (μM)	Pb^2+^ detected (%)	RSD (%)
Drinking water (bottled)	0.05	95.8	2.35
	0.1	99.5	1.81
	0.2	96.5	2.57
Tap water	0.05	102.3	2.95
	0.1	97.5	3.02
	0.2	95.5	2.13

## Conclusions

The electrochemical behaviour of Nb_2_CT_*x*_ and Nb_4_C_3_T_*x*_ was investigated to explore its potential in electrochemical applications. The Nb_4_C_3_T_*x*_ has demonstrated promising electrochemical performance and its electrochemical response is higher than Nb_2_CT_*x*_. The electrochemical detection capability of Nb_4_C_3_T_*x*_ towards Pb^2+^ ions has been investigated by stripping analysis at optimized conditions. Evident by the high sensitivity and good reproducibility, the large interlayer spacing of Nb_4_C_3_T_*x*_ can accommodate Pb^2+^ ions without destroying the layered structure of the electrode. The results showed that Nb_4_C_3_T_*x*_ can be used as an immobilization platform for sensitive detection of Pb^2+^ with wide linear range and detection limit of 12 nM. This work validates the potential application of Nb_4_C_3_T_*x*_ for the first time towards electrochemical sensing applications.

## Conflicts of interest

There are no conflicts to declare.

## Supplementary Material

RA-010-D0RA04377J-s001
