# Supplementary material for: Large interlayer spacing Nb4C3Tx (MXene) promotes the ultrasensitive electrochemical detection of Pb2+ on glassy carbon electrodes
Source: RSC Adv. 2020 Jun 29;10(41):24697–704. doi: 10.1039/d0ra04377j (PMC9055209; doi:10.1039/d0ra04377j)
Supplement: RA-010-D0RA04377J-s001 [file RA-010-D0RA04377J-s001.pdf]

Electronic Supplementary Material (ESI) for RSC Advances.
This journal is © The Royal Society of Chemistry 2020

Large interlayer spacing $Nb_4C_3T_x$ (MXene) promotes the ultrasensitive electrochemical detection of Pb^{2+} on glassy carbon electrode

P Abdul Rasheed[a], Ravi P Pandey[a], Tricia Gomez[a], Michael Naguib[b], Khaled A Mahmoud[a]*

[a]Qatar Environment and Energy Research Institute (QEERI), Hamad Bin Khalifa University (HBKU), Qatar Foundation, P.O. Box 34110, Doha, Qatar

[b]Department of Physics and engineering physics, Tulane University, New Orleans, LA, USA

* To whom all correspondence should be addressed:
E-mail: kmahmoud@hbku.edu.qa, Fax: +974 445441528, Phone: +974 44541694

Supporting Information

1. Synthesis of Nb_2AlC and Nb_4AlC_3 MAX phases

Powders of niobium (Alfa Aesar, 99.98%, -325 mesh), aluminum (Alfa Aesar, 99.9%, -325 mesh), and graphite C(Alfa Aesar, 99%, 7-11 micron) were mixed in ratios of 2Nb:1.3Al:1C for Nb_2AlC and 4Nb:1.5Al:2.7C for Nb_4AlC_3 were mixed for 3h at 56 rpm in a Turbula T2F mixer with yttria-stabilized zirconia balls as mixing media. After mixing, the Nb_2AlC powder was furnaced for 4 h at 1600°C with a 10 °C heating rate in a tube furnace under flowing argon. For Nb_4AlC_3, powders were pressed into ~10 g pellets and furnaced at 1700 °C for 1 h with a 10°C heating rate in a tube furnace under flowing argon. After furnacing, the products were ground to -400 mesh before etching. [1, 2]

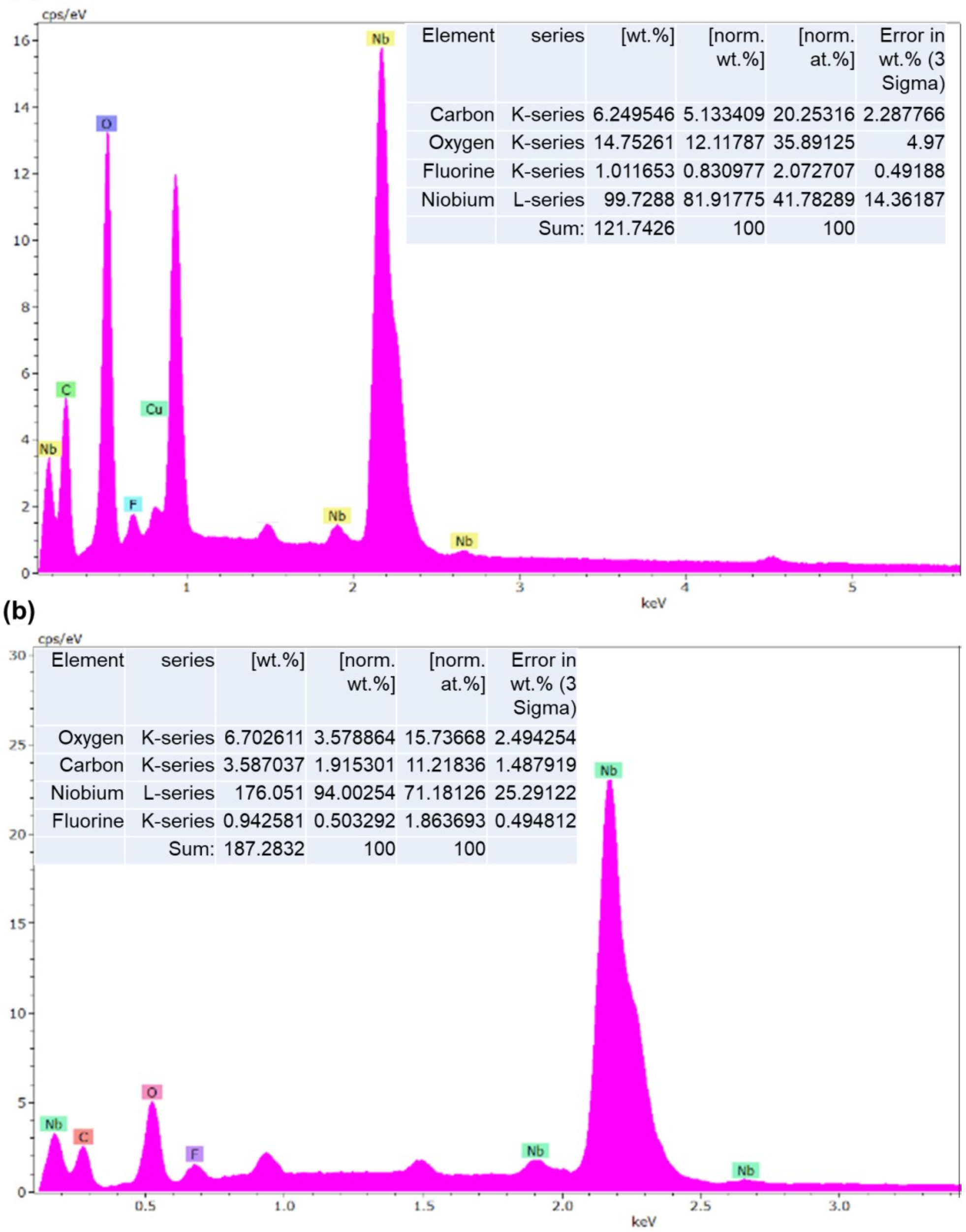

(a)

Element	series	[wt.%]	[norm. wt.%]	[norm. at.%]	Error in wt.% (3 Sigma)
Carbon	K-series	6.249546	5.133409	20.25316	2.287766
Oxygen	K-series	14.75261	12.11787	35.89125	4.97
Fluorine	K-series	1.011653	0.830977	2.072707	0.49188
Niobium	L-series	99.7288	81.91775	41.78289	14.36187
	Sum:	121.7426	100	100	

(b)

Element	series	[wt.%]	[norm. wt.%]	[norm. at.%]	Error in wt.% (3 Sigma)
Oxygen	K-series	6.702611	3.578864	15.73668	2.494254
Carbon	K-series	3.587037	1.915301	11.21836	1.487919
Niobium	L-series	176.051	94.00254	71.18126	25.29122
Fluorine	K-series	0.942581	0.503292	1.863693	0.494812
	Sum:	187.2832	100	100	

Fig. S1. EDX data for (a) DL-Nb_2CT_x and (b) DL-$Nb_4C_3T_x$.

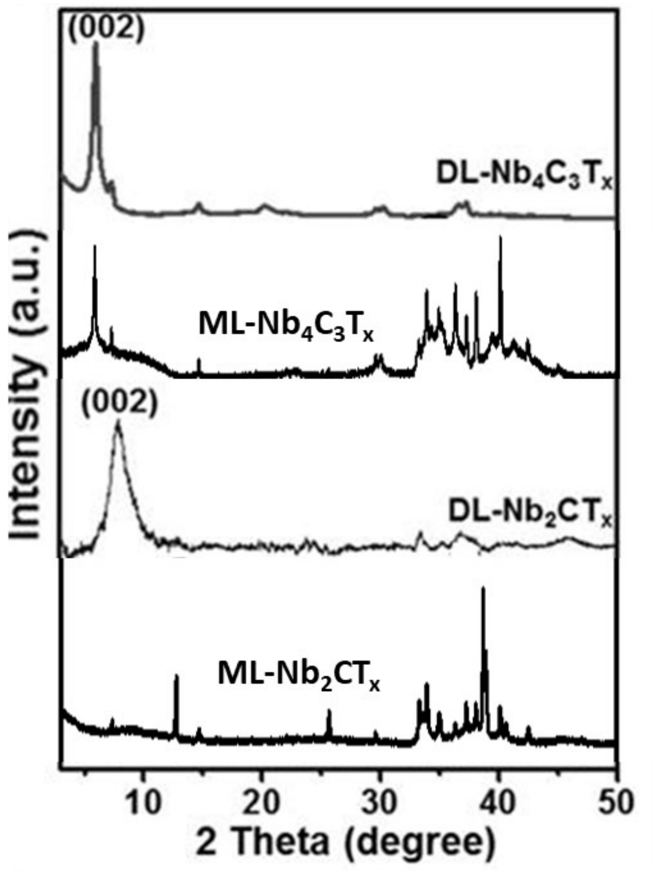

Fig. S2. XRD pattern of ML-Nb_2CT_x, DL-Nb_2CT_x, ML-$Nb_4C_3T_x$, and DL-$Nb_4C_3T_x$.

2. Calculation of the electrochemical active surface area using Randles–Sevcik equation

The Randles–Sevcik equation is $i_p = 2.69\times10^5\, n^{3/2}\, AD^{1/2}Cv^{1/2}$

Where i_p = current maximum in amps, n = number of electrons transferred in the redox event (usually 1), A = electrode area in cm^2, D = diffusion coefficient in cm^2/s, C = concentration in mol/cm^3 and v = scan rate in V/s.

The $n^{3/2}$ of 10mM of $K_3[Fe(CN)_6]$ is 1 and Diffusion coefficient, D is 7.6×10^{-6} cms^{-1}. The electrochemical surface area was calculated from the anodic peak current at the scan rate $100mVs^{-1}$.

From the above equation, the electrochemical active surface area was calculated as 0.574×10^{-3} cm^2 and 0.621×10^{-3} cm^2 for Nb_2CT_x and $Nb_4C_3T_x$ respectively.[3]

References

1. J. Yang, M. Naguib, M. Ghidiu, L.-M. Pan, J. Gu, J. Nanda, J. Halim, Y. Gogotsi and M. W. Barsoum, *Journal of the American Ceramic Society*, 2016, **99**, 660-666.
2. M. Naguib, J. Halim, J. Lu, K. M. Cook, L. Hultman, Y. Gogotsi and M. W. Barsoum, *Journal of the American Chemical Society*, 2013, **135**, 15966-15969.
3. P. V. Sarma, C. S. Tiwary, S. Radhakrishnan, P. M. Ajayan and M. M. Shaijumon, *Nanoscale*, 2018, **10**, 9516-9524.
